# Limonoids from the Fruits of *Khaya ivorensis*

**DOI:** 10.3390/molecules19033004

**Published:** 2014-03-07

**Authors:** Kai-Long Ji, Shang-Gao Liao, Xiao-Ling Zheng, Zhi Na, Hua-Bin Hu, Ping Zhang, You-Kai Xu

**Affiliations:** 1 CAS Key Laboratory of Tropical Plant Resource and Sustainable Use, Xishuangbanna Tropical Botanical Garden, Chinese Academy of Sciences, Menglun, Mengla, Yunnan 666303, China; 2 University of Chinese Academy of Sciences, Beijing 100049, China; 3 Engineering Research Center for the Development and Application of Ethnic Medicines and TCM, School of Pharmacy, Guiyang Medical College, 9 Beijing Road, Guiyang, Guizhou 550004, China

**Keywords:** *Khaya ivorensis*, limonoids, 14,15-didehydroruageanin A, 3-*O*-methylbutyryl- seneganolide A, cytotoxicity

## Abstract

Two new limonoids, namely 14,15-didehydroruageanin A (**1**) and 3-*O*-methyl- butyrylseneganolide A (**2**), were isolated from the fruits of *Khaya ivorensis* along with six known limonoids: seneganolide A (**3**), 1,3-dideacetylkhivorin (**4**), 7-deacetylkhivorin (**5**), 3-deacetylkhivorin (**6**), 1-deacetylkhivorin (**7**), and 3-deacetyl-7-oxokhivorin (**8**). All the compounds were evaluated for their cytotoxicity against five tumor cell lines.

## 1. Introduction

**Figure 1:**
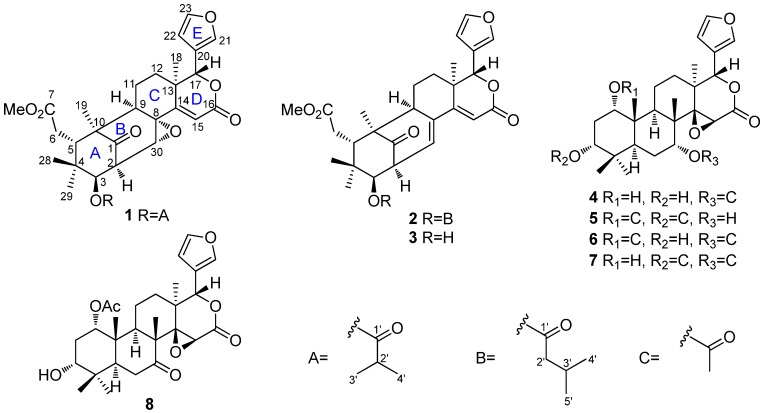
The chemical structures of compounds **1**–**8**.

Meliaceous limonoids, the major metabolites of the Meliaceae family, have attracted great interest in the natural products field due to their structural diversity and broad range of bioactivities [1,2]. *Khaya* A. Juss. (Meliaceae) is a genus of eight species mainly distributed in Africa and Madagascar [3]. *Khaya ivorensis* A. Chev. is a popular traditional African medicinal plant in this genus that is also cultivated in southern China [4,5,6]. Its ethanol stem bark extract has shown tissue toxicity, antimalarial and antiinflammatory activities [5]. Previous chemical investigation of *K. ivorensis* indicated that this plant was a good source of limonoids [6,7,8,9,10,11]. As a continuation of our studies of this medicinal plant in search of biologically significant secondary metabolites, the fruits of *K.* *ivorensis* were investigated. As a result, the EtOAc-soluble fraction of the ethanolic extract has produced after extensive column chromatography two new limonoids, 14,15-didehydroruageanin A (**1**), 3-*O*-methylbutyrylseneganolide A (**2**), and six known limonoids **3**–**8**, of which compounds **3**, **4**, **7**, and **8** were isolated in this plant for the first time (Figure 1). Their structures were established by NMR spectroscopic method and by comparison with literature data. The cytotoxic activities of all isolated compounds were also tested. Herein, the extraction, isolation, structure elucidation, and cytotoxic evaluation of these compounds are described.

## 2. Results and Discussion

**Figure 2:**
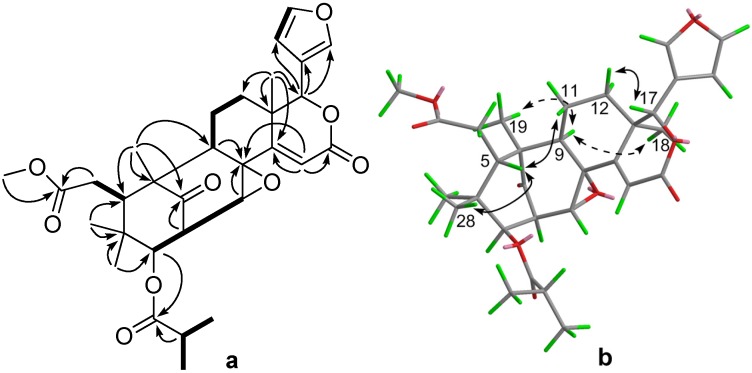
^1^H-^1^H COSY (bold) and selected HMBC correlations of **1** (**a**); Selected key ROESY correlations of **1** (**b**).

Compound **1**, was obtained as a white amorphous powder. Its HREIMS revealed a molecular ion peak at *m/z* 554.2501 (calcd. 554.2516), consistent with a molecular formula of C_31_H_38_O_9_ possessing 13 degrees of unsaturation. The ^1^H- and ^13^C-NMR (with DEPT) spectra (Table 1) showed, in addition to resonances for an isobutyryl [*δ*_H_ 1.23 (3H, d, *J* = 7.1 Hz), 1.26 (3H, d, *J* = 7.1 Hz), 2.81 (1H, m); *δ*_C_ 19.2 CH_3_, 19.6 CH_3_, 34.9 CH, 176.0 C] [12] and a methoxycarbonyl group [*δ*_H_ 3.65 (3H, s); *δ*_C_ 52.31 O*CH_3_*, 174.3 C], signals for a ketone (*δ*_C_ 214.8 C), four tertiary methyls (*δ*_H_ 0.79 s, 0.90 s, 1.13 s, 1.22 s; *δ*_C_ 16.1 CH_3_, 21.60 CH_3_, 21.7 CH_3_, 22.9 CH_3_), a furan ring [*δ*_H_ 6.70 (1H, br s), 7.70 (1H, br s), 7.88 (1H, br s); *δ*_C_ 111.4 CH, 121.1 C, 143.1 CH, 144.2 CH], and an *α*,*β*-unsaturated *δ*-lactone [*δ*_H_ 6.66 (1H, s); *δ*_C_ 119.5 CH, 162.0 C, 164.6 C]. An epoxy group was also identified by the NMR data [*δ*_H_ 4.51 (1H, d, *J* = 2.0); *δ*_C_ 61.5 C, 62.7 CH]. The above signals accounted for seven out of the 13 degrees of unsaturation and suggested the compound to thus be hexacyclic. These observations indicated that compound **1** was possibly a mexicanolide-type limonoid [12]. 2D-NMR correlation analysis (Figure 2) further confirmed this conclusion and allowed establishment of the full structure for **1**. In particular, HMBC correlation from H_2_-6 to the carboxyl at *δ*_C_ 174.3 located the methoxycarbonyl group at C-6, while that from H-3 to the isobutyryl carbonyl placed the isobutyryloxy group at C-3. The epoxy group was further revealed to be an 8,30-epoxy by ^1^H-^1^H COSY correlations between H-30/H-2/H-3 and HMBC correlations H-30/C-8, H-9/C-8, and H-15/C-8 (Figure 2).

**Table 1:** ^1^H- and ^13^C-NMR of **1** and **2**.

	1		2	
	*δ*_C_, type ^a^	*δ*_H_ (*J* in Hz) ^b^	*δ*_C_, type ^a^	*δ*_H_ (*J* in Hz) ^b^
1	214.8, s		214.8, s	
2	49.7, d	3.92 dd (9.3, 2.0)	49.9, d	3.94 ddd (9.0, 6.1, 1.3)
3	77.6, d	5.39 d (9.3)	79.1, d	5.14 d (9.2)
4	40.3, s		39.3, s	
5	42.6, d	3.67 d (2.0)	41.2, d	3.57 dd (7.2, 4.7)
6	33.6, t	2.44 m	33.5, t	2.55 m
7	174.3, s		174.5, s	
8	61.5, s		136.9, s	
9	56.3, d	2.11 br s	54.1, d	2.46 m
10	49.0, s		52.5, s	
11	21.59, t	*α* 1.22 m, *β* 1.65 m	22.1, t	*α* 1.81 m, *β* 1.66 m
12	33.2, t	*α* 1.26 m, *β* 2.11 m	32.9, t	*α* 1.19 m, *β* 1.95 m
13	39.7, s		38.0, s	
14	162.0, s		161.1, s	
15	119.5, d	6.66 s	113.3, d	6.57 s
16	164.6, s		165.3, s	
17	80.0, d	5.57 s	80.1, d	5.20 s
18	21.7, q	1.22 s	22.2, q	1.08 s
19	16.1, q	1.13 s	16.1, q	1.30 s
20	121.1, s		121.6, s	
21	143.1, d	7.88 br s	142.6, d	7.81 s
22	111.4, d	6.70 br s	111.3, d	6.66 d (1.0)
23	144.2, d	7.70 br s	144.1, d	7.67 t (1.6)
28	22.9, q	0.90 s	23.0, q	0.93 s
29	21.60, q	0.79 s	21.4, q	0.84 s
30	62.7, d	4.51 d (2.0)	129.8, d	6.55, dd (6.0, 2.9)
7-OMe	52.3, q	3.65 s	52.3, q	3.69 s
3-acyl-1'	176.0, s		172.7, s	
2'	34.9, d	2.81 m	43.4, t	2.35 m
3'	19.6, q	1.26 d (7.1)	26.0, d	2.19 dt (13.7, 6.8)
4'	19.2, q	1.23 d (7.1)	22.82, q	0.95 d (6.7)
5'			22.85, q	0.92 d (6.7)

^a^ Recorded at 150 MHz in pyridine-*d*_5_; ^b^ Recorded at 600 MHz in pyridine-*d*_5_.

The relative configuration of **1** was established by ROESY correlation analysis (Figure 2a). The ROESY correlations of H-9/H_3_-19 and H-9/H_3_-18 indicated that H-9 was *α*-oriented, while those of H-5/H-11*β*, H-5/H_3_-28, and H-17/H-11*β* showed H-5 and H-17 were *β*-oriented. The ROESY correlation of H_3_-29/H-3 suggested that H-3 was *α*-oriented. The close coupling constant of H-3 in **1** (*J* = 9.3 Hz) and 2'*R*-cipadesin A (*J* = 9.5 Hz) [13], whose structure has been confirmed by X-ray crystal analysis [14], indicated that both compounds shared the same stereochemistry for H-2 and H-3 and H-2 was assigned to be *α*-oriented. The 8,30-epoxy was then determined to be *α*-oriented on the basis of the small coupling constant of H-30 (*J* = 2.0 Hz) [13]. Therefore, the relative stereochemistry of **1** was established as shown in Figure 1 and it was named 14,15-didehydroruageanin A.

**Figure 3:**
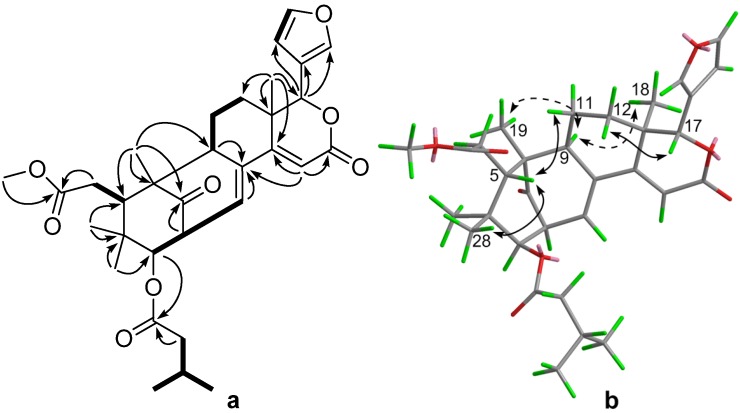
^1^H-^1^H COSY (bold) and selected HMBC correlations of **2** (**a**); Selected key ROESY correlations of **2** (**b**).

Compound **2**, was obtained as a white amorphous powder. Its molecular formula C_32_H_40_O_8_ was established by the HREIMS ion peak at *m/z* 552.2715 (calcd. 552.2723). Analysis of its ^1^H- and ^13^C-NMR data (Table 1) showed that **2** was also a mexicanolide-type compound, very similar to the known compound seneganolide A [15]. The only differences in the NMR data of the two compounds was the upfield-shift of the signals for C-3 (*δ*_C_ from 80.2 to 79.1) and the appearance of additional signals for a 3-methylbutyryl group [*δ*_H_ 0.92 (3H, d, *J* = 6.7 Hz), 0.95 (3H, d, *J* = 6.7 Hz), 2.19 (1H, dt, *J* = 13.7, 6.8 Hz), 2.35 (2H, m); *δ*_C_ 22.82 CH_3_, 22.85 CH_3_, 26.0 CH, 43.4 CH_2_, 172.7 C], which suggested that compound **2** was the 3-*O*-methylbutyryl derivative of seneganolide A. ^1^H-^1^H COSY correlations H-4'/H-3'/H-2' and the HMBC correlation H-3/C-1' (Figure 3a) further confirmed this conclusion. ROESY correlation analysis (Figure 3b) also supported that both compounds shared the same stereochemistry. Thus, the relative structure of **2** was elucidated as shown in Figure 1 and it was named 3-*O*-methylbutyrylseneganolide A.

Six known limonoids were also isolated and identified by spectroscopic methods to be seneganolide A (**3**) [15], 1,3-dideacetylkhivorin (**4**) [16], 7-deacetylkhivorin (**5**) [7], 3-deacetylkhivorin (**6**) [11], 1-deacetylkhivorin (**7**) [17], and 3-deacetyl-7-oxokhivorin (**8**) [18]. Among these known limonoids, compounds **3**, **4**, **7**, and **8** were obtained from *K. ivorensis* for the first time.

Limonoids **1**–**8** were all evaluated for their cytotoxic activities against five human tumor cell lines: myeloid leukemia (HL-60), hepatocellular carcinoma (SMMC-7721), lung cancer (A-549), breast cancer (MCF-7), and colon cancer (SW480). However, only compounds **2**, **3**, and **4** exhibited cytotoxicity against certain tumor cell lines with the IC_50_ values in the range of 21.1–39.5 μM (Table 2). 

**Table 2:** The cytotoxicity (IC_50_ μM) of isolated compounds **1**–**8**.

Compound	HL-60	SMMC-7721	A-549	MCF-7	SW480
**1**	>40	>40	>40	>40	>40
**2**	>40	>40	37.3	>40	>40
**3**	21.2	21.1	23.8	>40	32.6
**4**	>40	>40	39.5	26.1	>40
**5**	>40	>40	>40	>40	>40
**6**	>40	>40	>40	>40	>40
**7**	>40	>40	>40	>40	>40
**8**	>40	>40	>40	>40	>40
Cisplatin ^a^	1.1	4.5	6.6	13.1	11.1

^a^ Positive control.

## 3. Experimental

### 3.1. General Information

Optical rotations were obtained with a JASCO P-1020 polarimeter. UV spectra were measured with a Shimadzu UV 2401PC. IR spectra (KBr) were determined on a Bruker Tensor-27 infrared spectrophotometer. 1D- and 2D-NMR spectra were recorded on Bruker AM-400, Bruker DRX-500 and Avance III 600 spectrometers with TMS as an internal standard (Karlsruhe, Germany). ESIMS and HREIMS recorded on a Xevo TQ-S mass spectrometer (Manchester, UK) and a Waters AutoSpec Premier P776 instrument (Milford, CT, USA), respectively. Semi-preparative HPLC was carried out using a Waters system (Milford, CT, USA) consisting of a 600 pump and a 2996 Photodiode Array Detector. Silica gel (200–300 mesh, Qingdao Marine Chemical Factory, Qingdao, China), Sephadex LH-20 gel (40–70 μM, Amersham Pharmacia Biotech AB, Uppsala, Sweden), and MCI gel (CHP20/P120, 75–150 μM, high porous polymer, Mitsubishi Chemical Corporation, Japan) were used for column chromatography (CC).

### 3.2. Plant Material

The fruits of *K.* *ivorensis* were collected from Xishuangbanna Tropical Botanical Garden (XTBG), Chinese Academy of Science (CAS), Mengla Country, Yunnan Province, People’s Republic of China, in July 2013 and were identified by one of the authors (Y.-K. Xu). A voucher specimen (No. 028765) was deposited in the herbarium of XTBG.

### 3.3. Extraction and Isolation

The air-dried and powdered fruits of *K. ivoremsis* (4.0 kg) were extracted three times (each for 7 days) with EtOH–H_2_O (95/5, *v/v*, 30 L) at room temperature. Removal of solvent from the combined extracts under vacuum afforded a crude residue (200 g). The residue was then suspended in H_2_O and partitioned with EtOAc (60 g). The EtOAc-soluble fraction was subjected to silica gel CC (petroleum ether-acetone from 1/0 to 0/1, *v/v*) to produce four fractions (1–4). Fraction 1 (5.0 g) was subjected to MCI gel CC (MeOH–H_2_O from 1/6 to 1/1, *v/v*) to give **4** (12 mg), **5** (5 mg), and **6** (7 mg). Fraction 2 (12.5 g) was subjected to silica gel CC to obtain **3** (600 mg) and sub-fraction 2A (10 mg). Fraction 2A was then purified by semi-preparative HPLC (MeOH–H_2_O from 2/3 to 7/3, *v/v*) to yield **2 **(4 mg). Fraction 3 (2.0 g) was separated by Sephadex LH-20 CC eluted with MeOH–H_2_O (from 1/4 to 1/1, *v/v*) and further purified by semi-preparative HPLC to give **1** (3 mg). Fraction 4 (12.0 g) was applied to a silica gel column and eluted with petroleum ether-acetone (from 1/9 to 1/1, *v/v*) to give **7** (800 mg) and a sub-fraction 4A (50 mg), purification of which by Sephadex LH-20 CC (MeOH–H_2_O from 3/7 to 3/2, *v/v*) and semi-preparative HPLC (CH_3_CN–H_2_O from 2/3 to 7/3, *v/v*) yielded **8** (4 mg).

### 3.4. Spectral Data

*14,15-Didehydroruageanin* A (**1**). White amorphous powder; [*α*]^20.5^_D_ +9.3 (*c* 0.045, MeOH); UV (MeOH) *λ*_max_ nm (log *ε*) 204.8 (4.23); IR (KBr) *ν*_max_ (cm^−1^): 2972, 2931, 1732, 1634, 1460, 1384, 1271, 1197, 1114, 1028; ^1^H- and ^13^C-NMR data, see Table 1; positive ESIMS *m/z* 555 [M+H]^+^; HREIMS *m/z* 554.2501 M (calcd for C_31_H_3__8_O_9_, 554.2516).

*3-O-Methylbutyrylseneganolide* A (**2**). White amorphous powder; [*α*]^15.4^_D_ +51.0 (*c* 0.076, MeOH); UV (MeOH) *λ*_max_ nm (log *ε*) 203 (4.08), 282.4 (3.89); IR (KBr) *ν*_max_ (cm^−1^): 2961, 1630, 1463, 1384, 1295, 1256, 1167, 1119, 1107, 1028, 1001; ^1^H- and ^13^C-NMR data, see Table 1; positive ESIMS *m/z* 553 [M+H]^+^; HREIMS *m/z* 552.2715 M (calcd for C_32_H_40_O_8_, 552.2723).

### 3.5. Cytotoxicity Assay

The MTT method [19] was used for assessing the cytotoxicity of all isolated compounds against the five tumor cell lines (HL-60 human myeloid leukemia, SMMC-7721 hepatocellular carcinoma, A-549 lung cancer, MCF-7 breast cancer, and SW480 colon cancer) with cisplatin as the positive control.

## 4. Conclusions

In summary, three mexicanolide-type limonoids, 14,15-didehydroruageanin A (**1**), 3-*O*-methylbutyrylseneganolide A (**2**), and seneganolide A (**3**), along with five known d-seco limonoids, 1,3-dideacetylkhivorin (**4**), 7-deacetylkhivorin (**5**), 3-deacetylkhivorin (**6**), 1-deacetylkhivorin (**7**) and 3-deacetyl-7-oxokhivorin (**8**), were isolated from the fruits of *Khaya ivorensis*. Among these compounds, **1** and **2** were new compounds; and compounds **3**, **4**, **7**, and **8** were obtained from this plant for the first time. The cytotoxicity evaluation showed that only compounds **2**, **3**, and **4** exhibited cytotoxicity against certain tumor cell lines.

## Supplementary Materials

Supplementary materials can be accessed at: http://www.mdpi.com/1420-3049/19/3/3004/s1.
