# Supplementary material for: Limonoids from the Fruits of Khaya ivorensis
Source: Molecules. 2014 Mar 7;19(3):3004–11. doi: 10.3390/molecules19033004 (PMC6271042; doi:10.3390/molecules19033004)
Supplement: Supplementary file 1 [file molecules-19-03004-s001.pdf]

Supporting Information

List of Figures

Figure S1. ^{1}H-NMR spectrum of 14,15-didehydroruageanin A (**1**) at 600 MHz in pyridine-d_5

Figure S2. ^{13}C-NMR spectrum of 14,15-didehydroruageanin A (**1**) at 150 MHz in pyridine-d_5

Figure S3. HSQC spectrum of 14,15-didehydroruageanin A (**1**) at 600/150 MHz in pyridine-d_5

Figure S4. HMBC spectrum of 14,15-didehydroruageanin A (**1**) at 600/150 MHz in pyridine-d_5

Figure S5. COSY spectrum of 14,15-didehydroruageanin A (**1**) at 600 MHz in pyridine-d_5

Figure S6. ROESY spectrum of 14,15-didehydroruageanin A (**1**) at 600 MHz in pyridine-d_5

Figure S7. HREI-MS spectrum of 14,15-didehydroruageanin A (**1**)

Figure S8. UV spectrum of 14,15-didehydroruageanin A (**1**)

Figure S9. IR spectrum of 14,15-didehydroruageanin A (**1**)

Figure S10. Optical rotation measurement of 14,15-didehydroruageanin A (**1**)

Figure S11. ^{1}H-NMR spectrum of 3-*O*-methylbutyrylseneganolide A (**2**) at 600 MHz in pyridine-d_5

Figure S12. ^{13}C-NMR spectrum of 3-*O*-methylbutyrylseneganolide A (**2**) at 150 MHz in pyridine-d_5

Figure S13. HSQC spectrum of 3-*O*-methylbutyrylseneganolide A (**2**) at 600/150 MHz in pyridine-d_5

Figure S14. HMBC spectrum of 3-*O*-methylbutyrylseneganolide A (**2**) at 600/150 MHz in pyridine-d_5

Figure S15. COSY spectrum of 3-*O*-methylbutyrylseneganolide A (**2**) at 600 MHz in pyridine-d_5

Figure S16. ROESY spectrum of 3-*O*-methylbutyrylseneganolide A (**2**) at 600 MHz in pyridine-d_5

Figure S17. HREI-MS spectrum of 3-*O*-methylbutyrylseneganolide A (**2**)

Figure S18. UV spectrum of 3-*O*-methylbutyrylseneganolide A (**2**)

Figure S19. IR spectrum of 3-*O*-methylbutyrylseneganolide A (**2**)

Figure S20. Optical rotation measurement of 3-*O*-methylbutyrylseneganolide A (**2**)

Figure S1. ^{1}H-NMR spectrum of 14,15-didehydroruageanin A (**1**) at 600 MHz in pyridine-d_5.

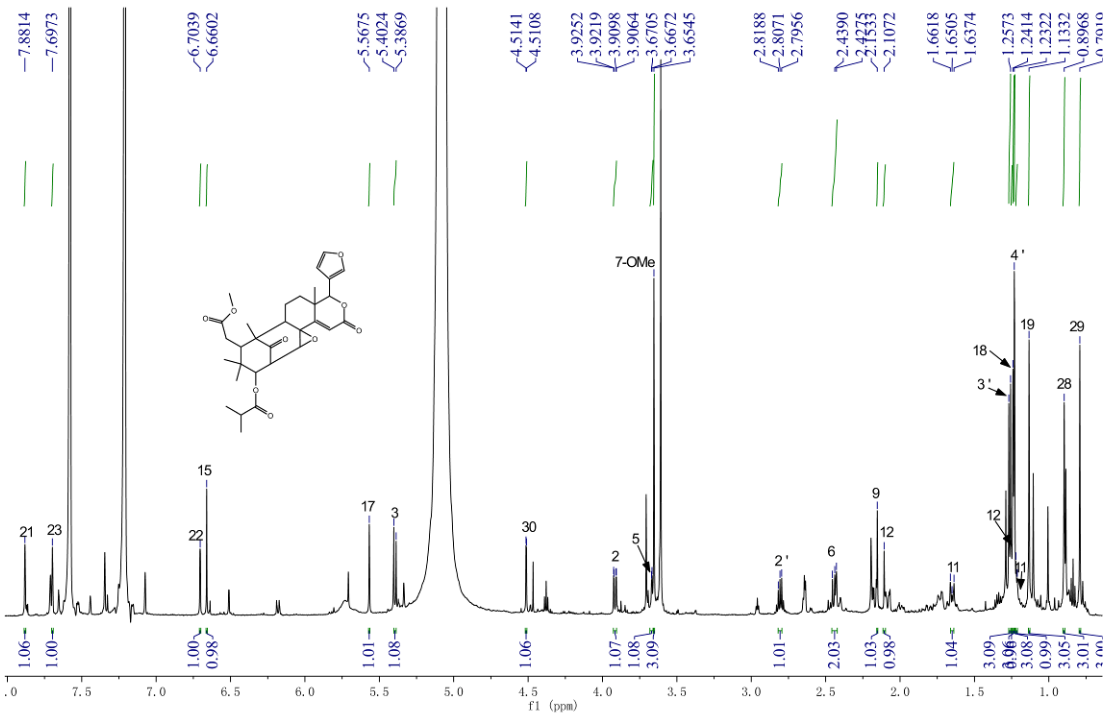

Figure S2. ^{13}C-NMR spectrum of 14,15-didehydroruageanin A (**1**) at 150 MHz in pyridine-d_5.

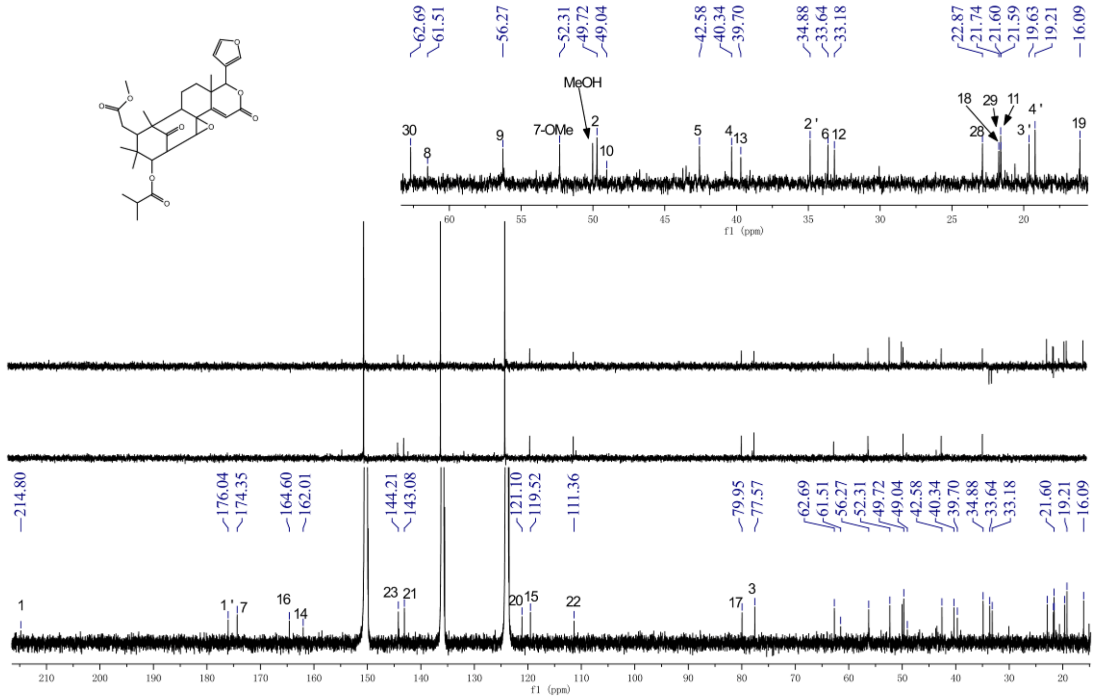

Figure S3. HSQC spectrum of 14,15-didehydroruageanin A (**1**) at 600/150 MHz in pyridine-d_5.

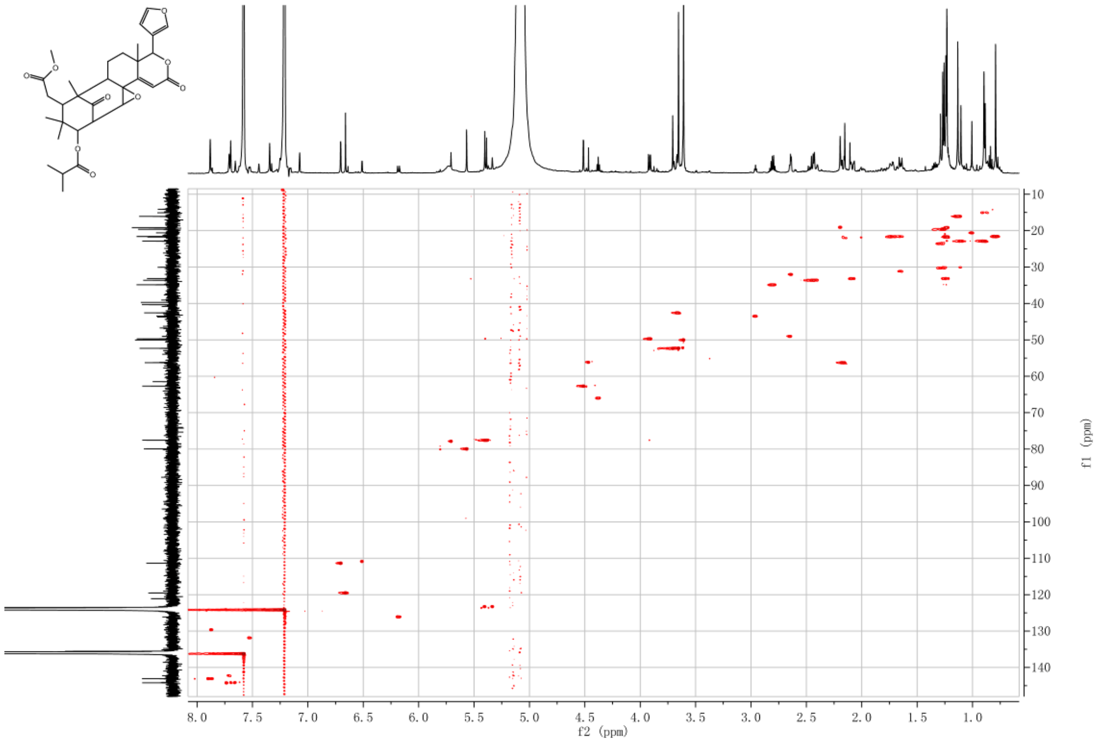

Figure S4. HMBC spectrum of 14,15-didehydroruageanin A (**1**) at 600/150 MHz in pyridine-d_5.

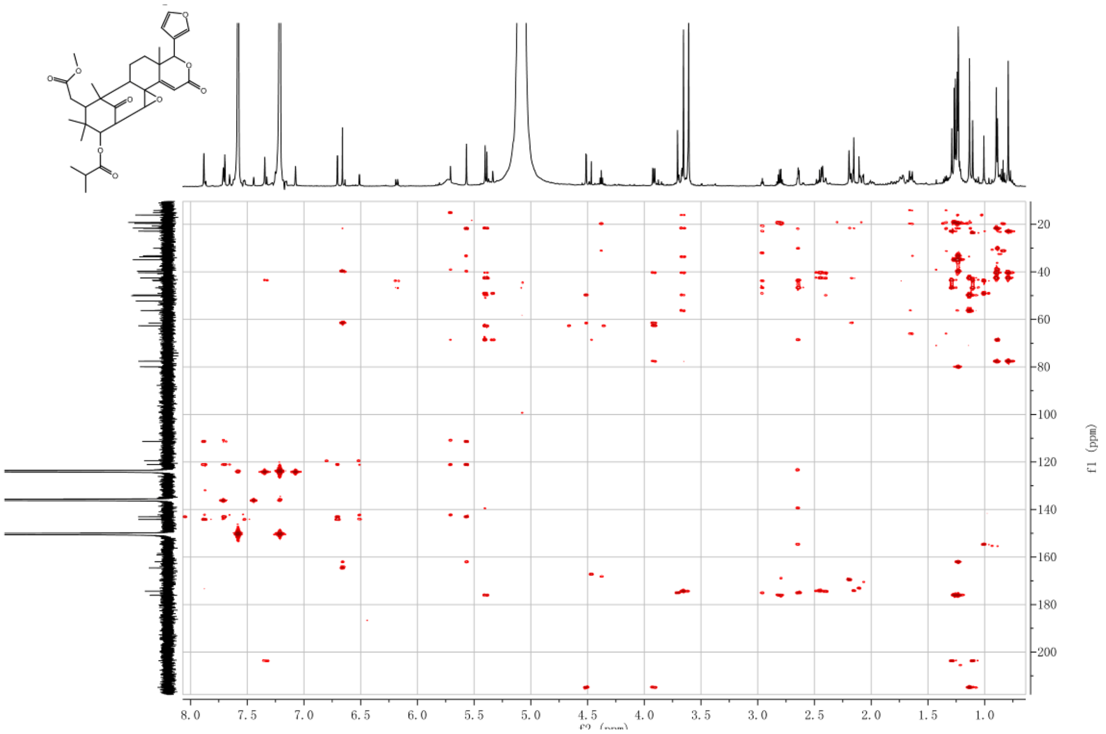

Figure S5. COSY spectrum of 14,15-didehydroruageanin A (**1**) at 600 MHz in pyridine-d_5.

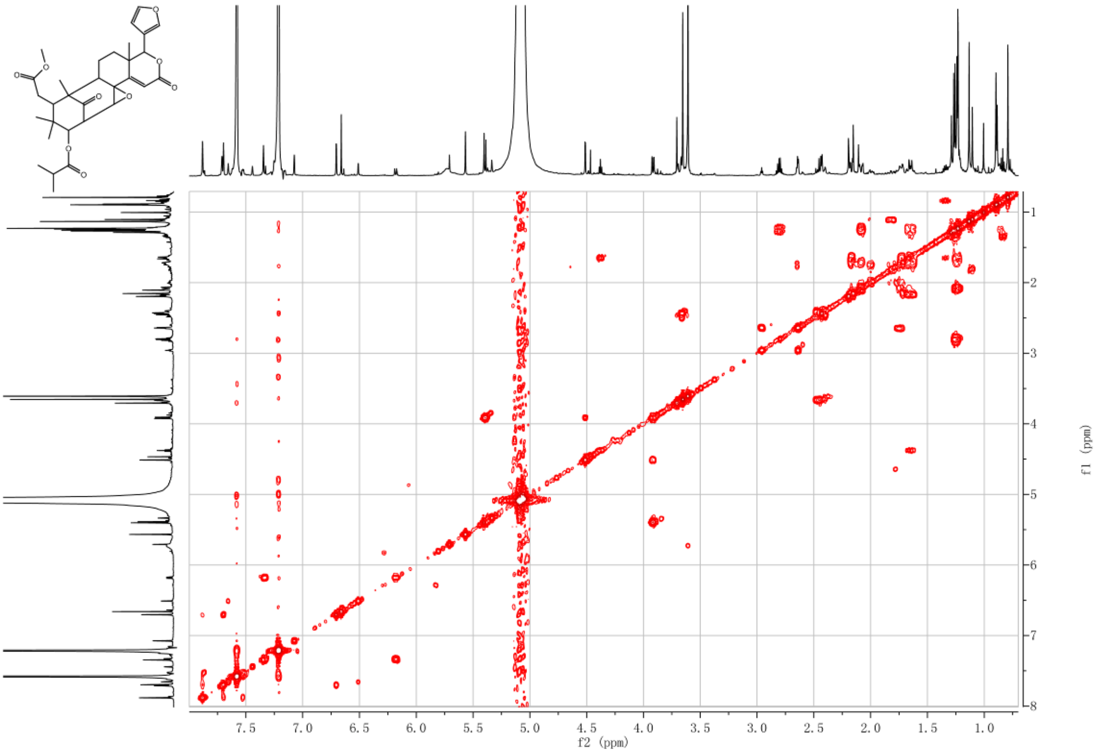

Figure S6. ROESY spectrum of 14,15-didehydroruageanin A (**1**) at 600 MHz in pyridine-d_5.

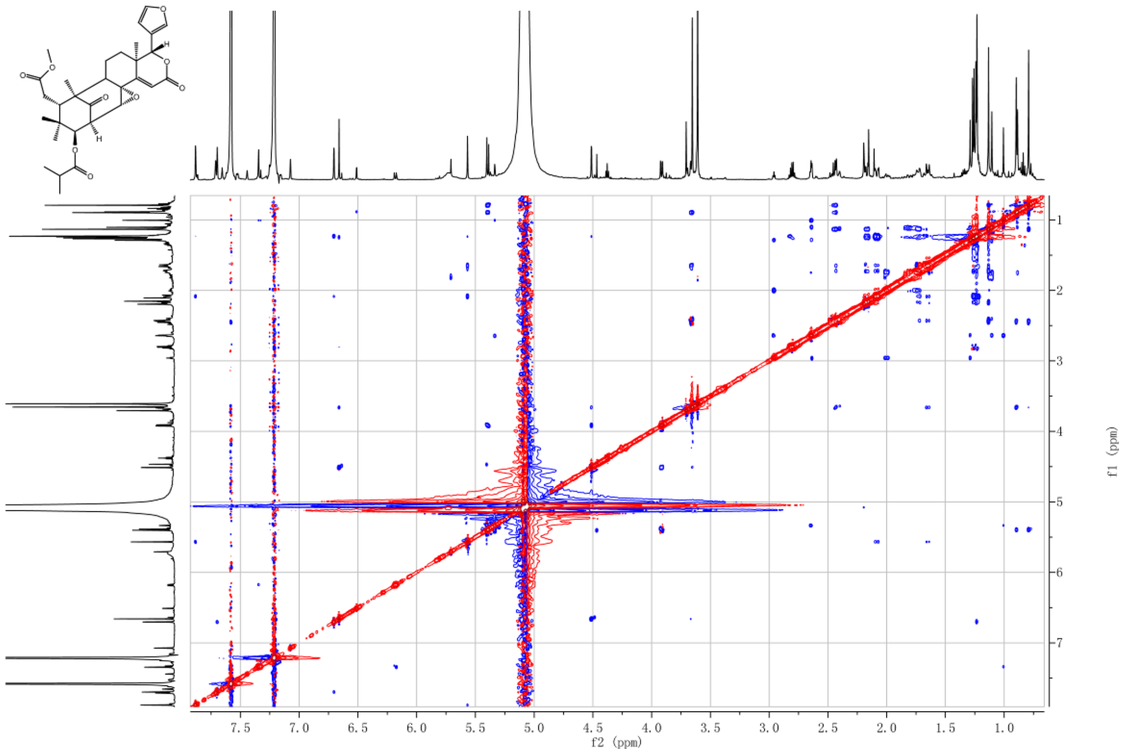

Figure S7. HREI-MS spectrum of 14,15-didehydroruageanin A (**1**).

Elemental Composition Report

Single Mass Analysis
Tolerance = 133.0 mDa / DBE: min = -10.0, max = 120.0
Selected filters: None

Monoisotopic Mass, Odd and Even Electron Ions
21 formula(e) evaluated with 9 results within limits (up to 51 closest results for each mass)
Elements Used:
C: 0-200 H: 0-400 O: 7-9

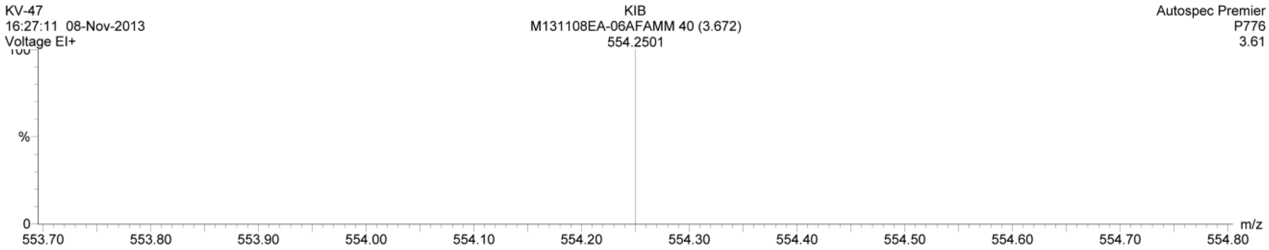

Minimum:				-10.0		
Maximum:		133.0	10.0	120.0		
Mass	Calc. Mass	mDa	PPM	DBE	i-FIT	Formula
554.2501	554.2516	-1.5	-2.7	13.0	166.3	C31 H38 O9
	554.2305	19.6	35.4	18.0	162.1	C34 H34 O7
	554.2880	-37.9	-68.4	12.0	173.5	C32 H42 O8
	554.1941	56.0	101.0	19.0	155.2	C33 H30 O8
	554.3244	-74.3	-134.1	11.0	181.0	C33 H46 O7
	554.1577	92.4	166.7	20.0	148.6	C32 H26 O9
	554.3455	-95.4	-172.1	6.0	185.6	C30 H50 O9
	554.1366	113.5	204.8	25.0	144.8	C35 H22 O7
	554.3819	-131.8	-237.8	5.0	193.5	C31 H54 O8

Figure S8. UV spectrum (MeOH) of 14,15-didehydroruageanin A (**1**).

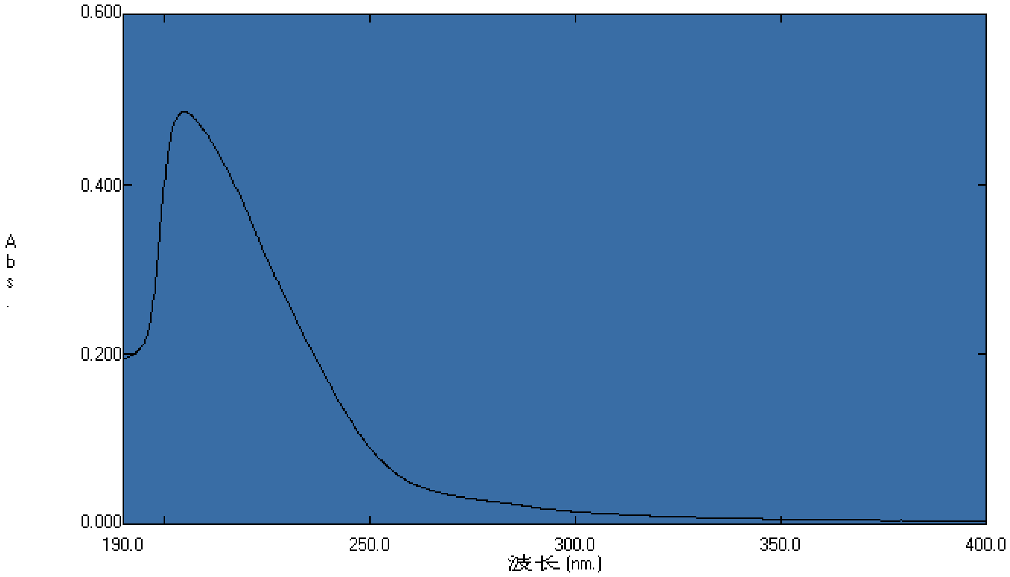

Figure S9. IR spectrum of 14,15-didehydroruageanin A (**1**).

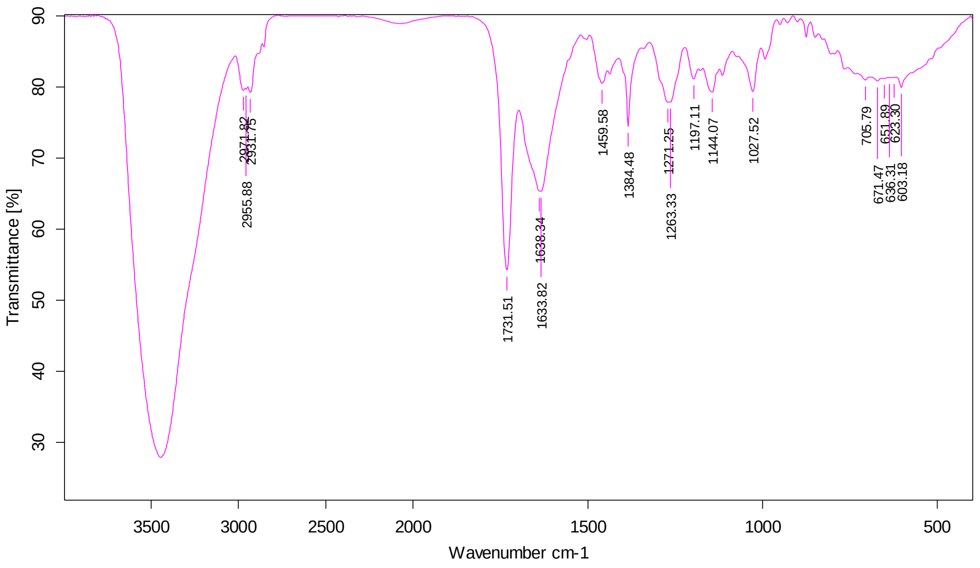

Sample : KV-47	Frequency Range : 399.246 - 3996.32	Measured on : 18/11/2013	
Technique : KBr压片	Resolution : 4	Instrument : Tensor27	Sample Scans : 16
Customer : 131118IR1	Zerofilling : 2	Acquisition : Double Sided,Forv	

Figure S10. Optical rotation measurement of 14,15-didehydroruageanin A (**1**).

11 (1/3)	Specific O.R.	5.333	20.5	0	Mon Nov 18 18:56:32 2013	0.00045 g/mL MeOH
11 (2/3)	Specific O.R.	12.444	20.5	0	Mon Nov 18 18:56:45 2013	0.00045 g/mL MeOH
11 (3/3)	Specific O.R.	7.556	20.5	0	Mon Nov 18 18:56:59 2013	0.00045 g/mL MeOH
12 (1/3)	Specific O.R.	5.778	20.5	0	Mon Nov 18 18:57:53 2013	0.00045 g/mL MeOH
12 (2/3)	Specific O.R.	10.222	20.5	0	Mon Nov 18 18:58:06 2013	0.00045 g/mL MeOH
12 (3/3)	Specific O.R.	4.000	20.5	0	Mon Nov 18 18:58:20 2013	0.00045 g/mL MeOH
13 (1/3)	Specific O.R.	9.333	20.5	0	Mon Nov 18 18:58:38 2013	0.00045 g/mL MeOH
13 (2/3)	Specific O.R.	14.667	20.5	0	Mon Nov 18 18:58:51 2013	0.00045 g/mL MeOH
13 (3/3)	Specific O.R.	14.222	20.5	0	Mon Nov 18 18:59:05 2013	0.00045 g/mL MeOH

Figure S11. ^{1}H-NMR spectrum of 3-*O*-methylbutyrylseneganolide A (**2**) at 600 MHz in pyridine-d_5.

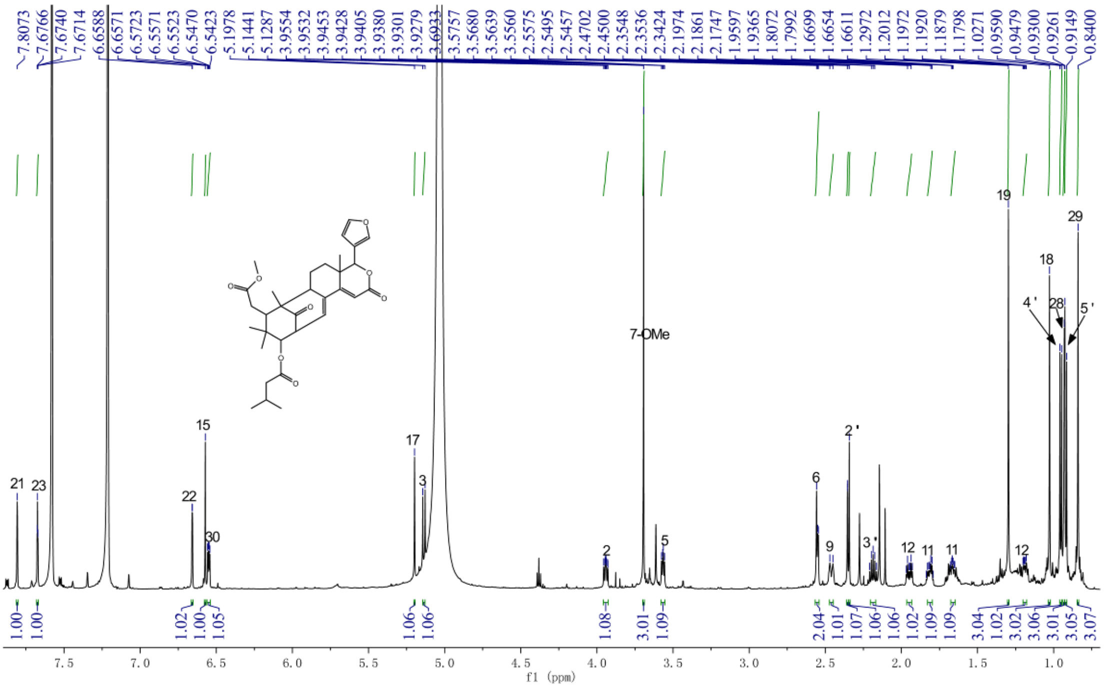

Figure S12. ^{13}C-NMR spectrum of 3-*O*-methylbutyrylseneganolide A (**2**) at 150 MHz in pyridine-d_5.

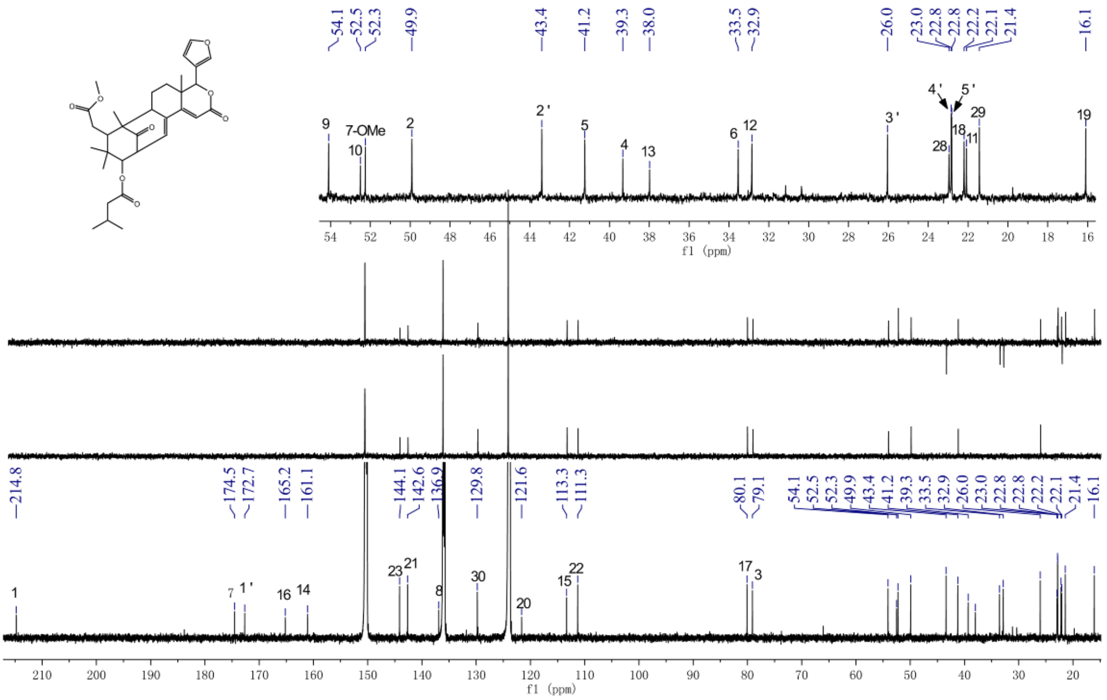

Figure S13. HSQC spectrum of 3-*O*-methylbutyrylseneganolide A (**2**) at 600/150 MHz in pyridine-d_5.

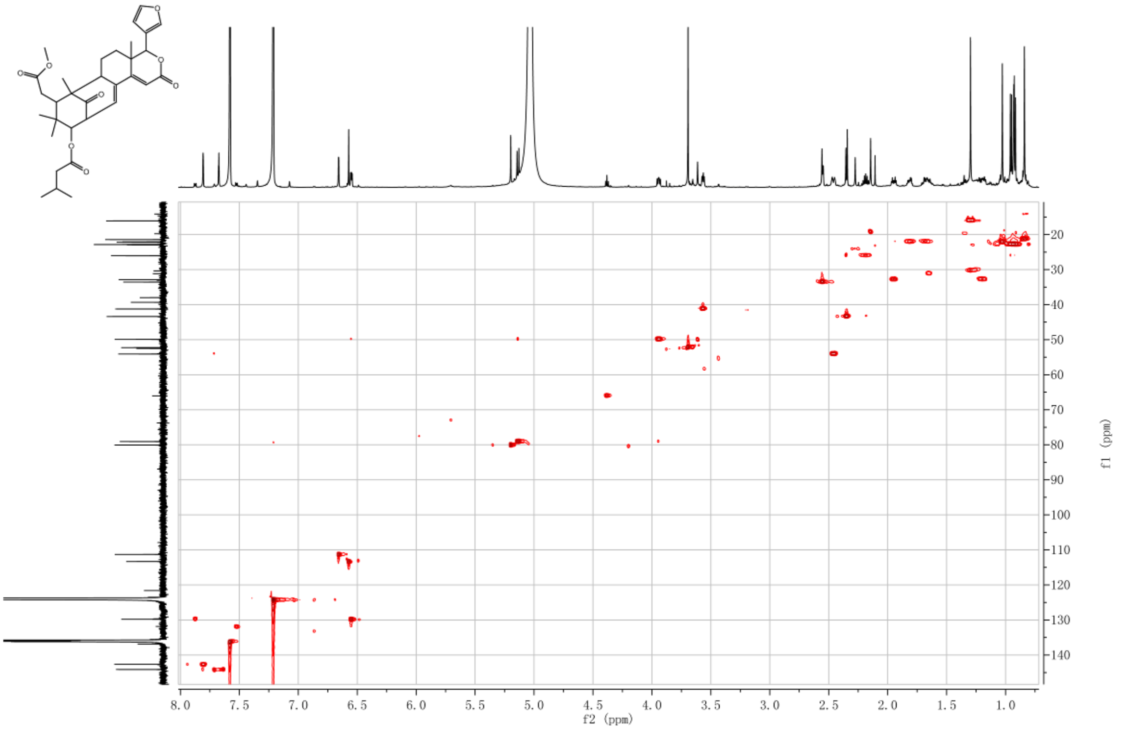

Figure S14. HMBC spectrum of 3-*O*-methylbutyrylseneganolide A (**2**) at 600/150 MHz in pyridine-d_5.

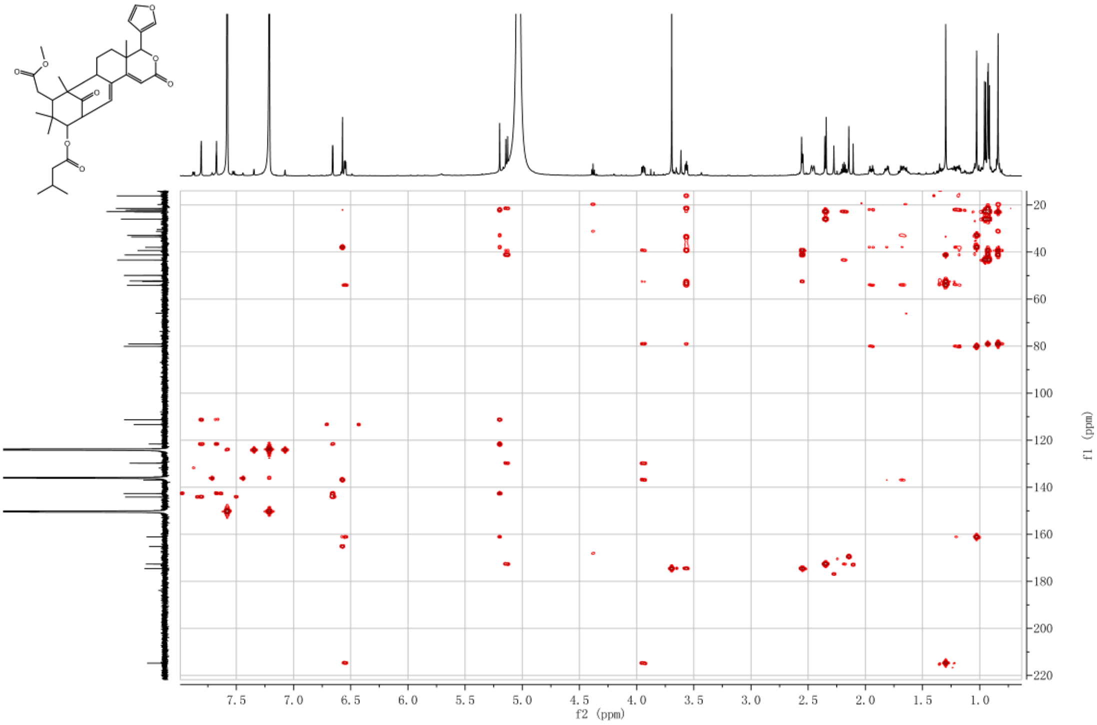

Figure S15. COSY spectrum of 3-*O*-methylbutyrylseneganolide A (**2**) at 600 MHz in pyridine-d_5.

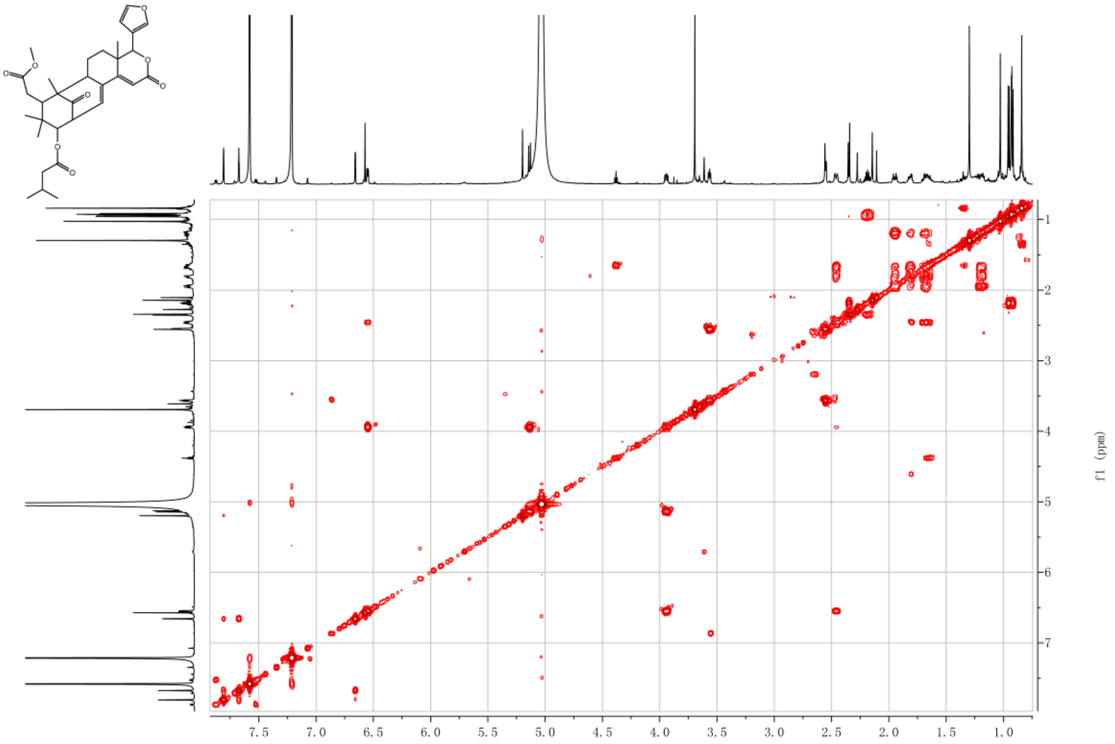

Figure S16. ROESY spectrum of 3-*O*-methylbutyrylseneganolide A (**2**) at 600 MHz in pyridine-d_5.

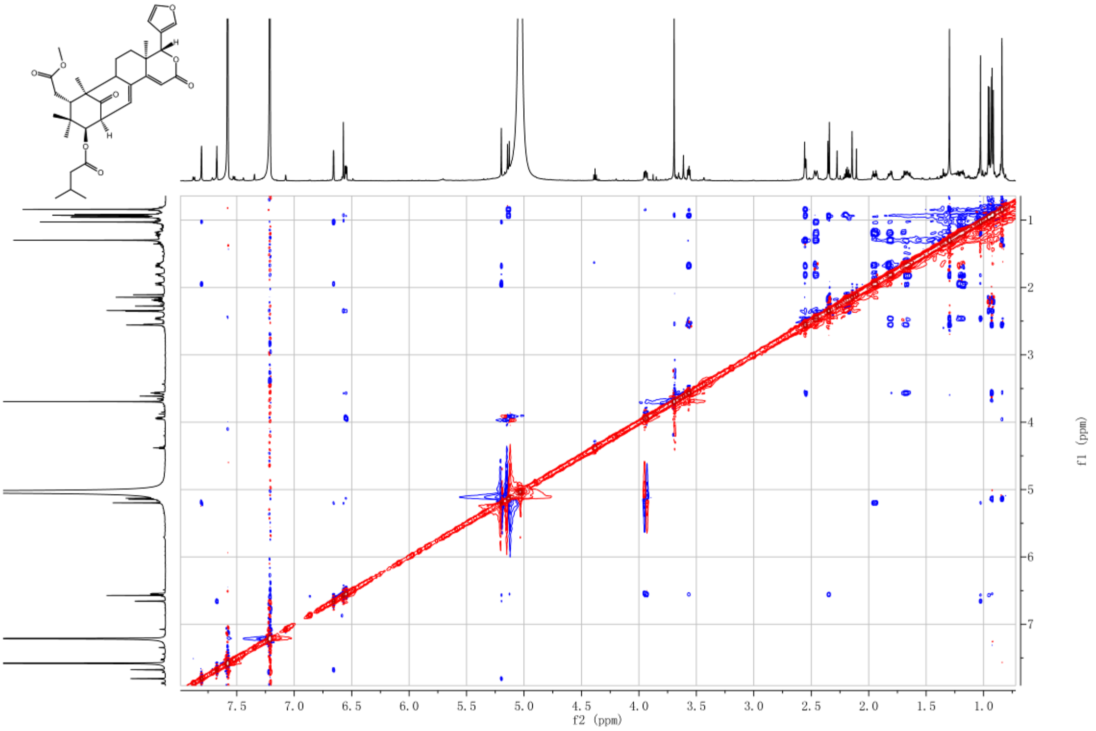

Figure S17. HREI-MS spectrum of 3-*O*-methylbutyrylseneganolide A (**2**).

Elemental Composition Report

Single Mass Analysis
Tolerance = 10.0 PPM / DBE: min = -10.0, max = 120.0
Selected filters: None

Monoisotopic Mass, Odd and Even Electron Ions
21 formula(e) evaluated with 1 results within limits (up to 51 closest results for each mass)
Elements Used:
C: 0-200 H: 0-400 O: 7-9

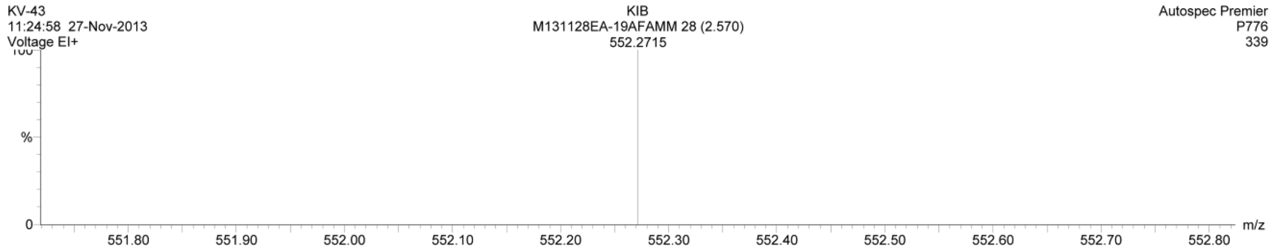

Minimum:				-10.0		
Maximum:		200.0	10.0	120.0		
Mass	Calc. Mass	mDa	PPM	DBE	i-FIT	Formula
552.2715	552.2723	-0.8	-1.4	13.0	5546188.0	C32 H40 O8

Figure S18. UV spectrum of 3-*O*-methylbutyrylseneganolide A (**2**).

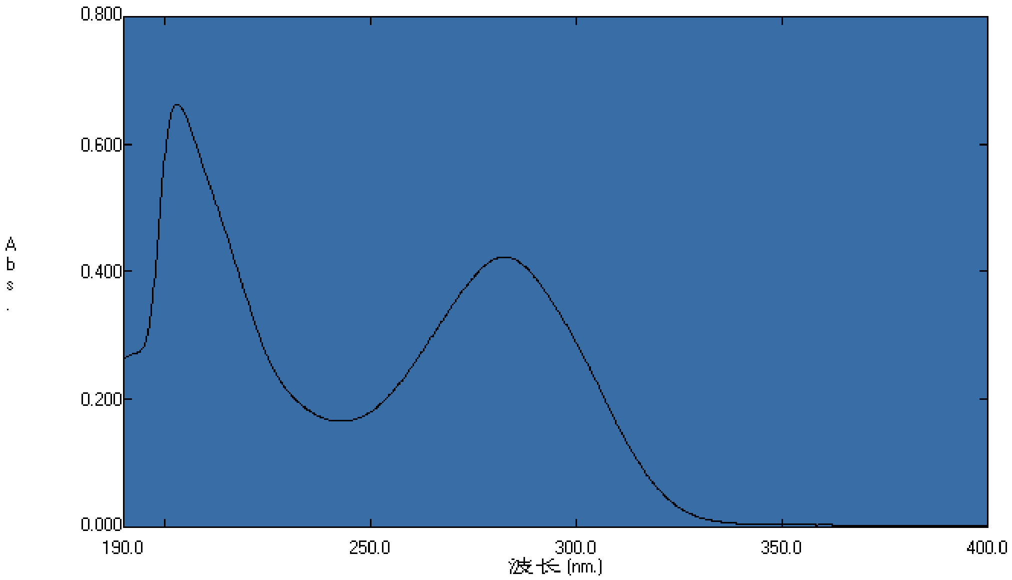

Figure S19. IR spectrum of 3-*O*-methylbutyrylseneganolide A (**2**).

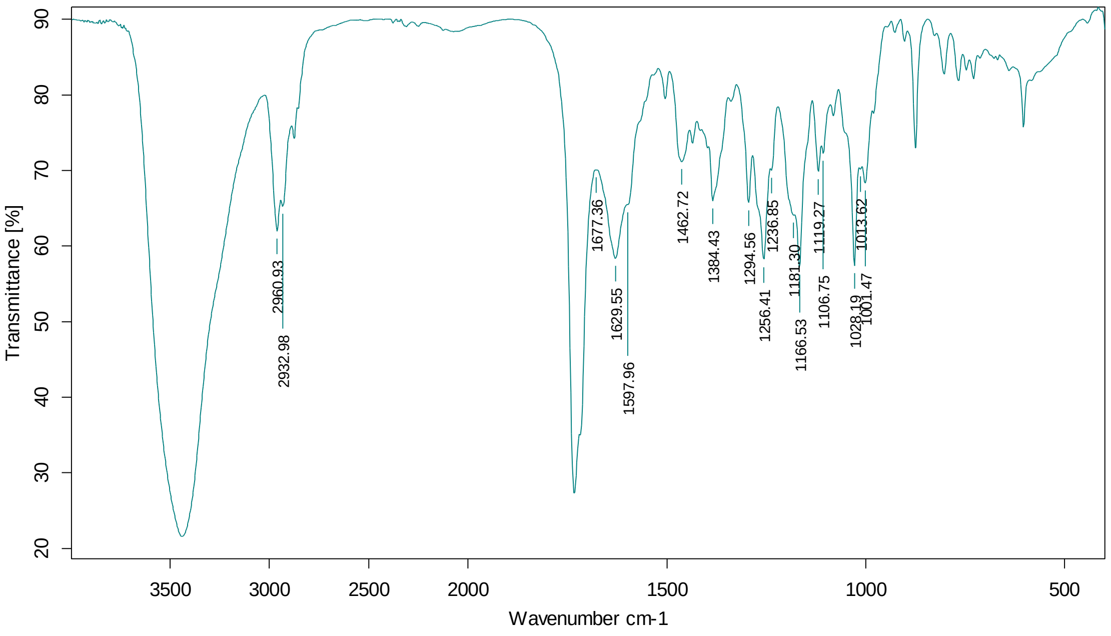

Sample : KV-43	Frequency Range : 399.246 - 3996.32	Measured on : 29/11/2013	
Technique : KBr压片	Resolution : 4	Instrument : Tensor27	Sample Scans : 16
Customer : 131202IR0	Zerofilling : 2	Acquisition : Double Sided,Forv	

Figure S20. Optical rotation measurement of 3-*O*-methylbutyrylseneganolide A (**2**).

1 (1/3)	Specific O.R.	51.579	15.5	0	Fri Nov 29 13:45:57 2013	0.00076 g/mL	MeOH
1 (2/3)	Specific O.R.	51.316	15.4	0	Fri Nov 29 13:46:11 2013	0.00076 g/mL	MeOH
1 (3/3)	Specific O.R.	50.000	15.4	0	Fri Nov 29 13:46:24 2013	0.00076 g/mL	MeOH
